# Exploring Trial Endpoints in Geographic Atrophy Based on Localized Functional Changes in Microperimetry and AI-Quantified OCT Biomarkers

**DOI:** 10.1167/iovs.67.1.22

**Published:** 2026-01-09

**Authors:** Klaudia Birner, Julia Mai, Daniela Boryshchuk, Florian Frommlet, Marie Louise Enzendorfer, Simon Schürer-Waldheim, Markus Gumpinger, Wolf-Dieter Vogl, Oliver Leingang, Stefan Sacu, Ursula Schmidt-Erfurth

**Affiliations:** 1 Department of Ophthalmology and Optometry, Medical University of Vienna, Vienna, Austria; 2 Center for Medical Data Science, Institute of Medical Statistics, Medical University of Vienna, Vienna, Austria; 3 RetInSight, Vienna, Austria

**Keywords:** age-related macular degeneration (AMD), geographic atrophy (GA)

## Abstract

**Purpose:**

The introduction of novel therapeutics for geographic atrophy (GA) highlights the need to define functional correlates of high-risk optical coherence tomography (OCT) biomarkers, in particular retinal-pigment-epithelium (RPE) loss and ellipsoid zone (EZ) loss. We conducted a pointwise structure/function correlation between OCT-based markers and retinal sensitivity (RS) in microperimetry (MP) in GA.

**Methods:**

Patients from the phase III OAKS clinical trial (NCT03525613) examined by OCT (Heidelberg Spectralis) and Macular Integrity Assessment (MAIA, iCare, and Centervue) MP in a 68-point grid were analyzed. Deep-learning-(DL)-based algorithms quantified RPE, EZ loss, and EZ thickness from OCT. Co-registration between MP and OCT was established between each MP stimulus and OCT B-scan location. A multivariable mixed effect model was implemented to identify RS for each OCT biomarker, accounting for eccentricity.

**Results:**

Six hundred seventy-eight study and fellow eyes of 406 patients with 41,925 MP points were included. Mean RS was 17 ± 7 decibel (dB), 9 ± 7 dB, and 2 ± 6 dB in intact retina, EZ, and RPE loss, respectively. Increased EZ thickness improved RS by 0.2 dB/µm (95% confidence interval [CI] = 0.2 to 0.2, *P* < 0.001). In areas of EZ loss, RS was significantly reduced compared to intact retina (−8 dB, 95% CI = −9 to −8]), whereas RPE loss decreased RS by −14 dB (95% CI = −15 to −14), accounting for eccentricity (all *P* < 0.001).

**Conclusions:**

A significant association between RS in MP and EZ and RPE loss on OCT was established using DL. A reliable quantitative structure/function association provides the base for developing functional endpoints in clinical care and approval of novel therapeutics for GA.

Progress in deep-learning (DL)-based assessment of disease activity on optical coherence tomography (OCT) in retinal diseases opens a novel diagnostic horizon.^1^ This development enables fast, precise, and objective quantification of pathognomonic biomarkers in age-related macular degeneration (AMD),^2^^,^^3^ which remains the leading cause of vision loss in adults over 50 years of age in developed countries.^4^ Geographic atrophy (GA), the late non-exudative stage of AMD, is characterized by loss of the retinal pigment epithelium (RPE) and thinning of the photoreceptor layer at the level of the ellipsoid zone (EZ).^5^ Conventional implementation of fundus autofluorescence (FAF) for GA diagnosis demonstrates that the three-dimensional display of outer retinal morphology on OCT allows for advanced monitoring of subclinical high-risk markers of GA at the level of the photoreceptors.^6^^,^^7^ Approval of complement inhibition as the first treatment for GA in 2023 was a therapeutic breakthrough, while highlighting challenges in defining functional trial endpoints in non-exudative AMD.^8^^,^^9^ The conventional use of best-corrected visual acuity (BCVA) to measure functional impairment is not well-suited to the parafoveal onset of GA lesions, whereas subfoveal GA leads to irreversible loss in BCVA, irrespective of GA lesion area changes.^10^ Therefore, improvement of functional assessment in GA, combined with a precise structural understanding of GA progression, is inevitable for defining clinically relevant therapeutic targets and for optimized patient management.^9^

Early studies demonstrated pronounced retinal sensitivity (RS) loss in areas with GA.^11^ RS measurement using microperimetry (MP) has gained recognition in recent literature, as it allows for comprehensive mapping of the entire macular region, which appears well-suited for functional monitoring of GA lesions.^12^^–^^14^ Still, despite growing interest in MP, its optimal implementation in GA is not yet fully understood. Recent studies include correlations between GA size and mean RS, perilesional sensitivity,^15^ number of scotomatous points,^11^^,^^13^ and localized changes with standard or high-density MP grids.^12^^,^^16^^,^^17^

We aimed to establish a precise topographical, pointwise structure/function correlation between RS changes in MP and the corresponding EZ and RPE loss quantified in OCT volumes using DL.

## Methods

### Patient Cohort and Inclusion Criteria

This post hoc analysis was performed on the baseline data from the phase III OAKS clinical trial (ClinicalTrials.gov, identifier: NCT03525613), a prospective, multicenter, randomized, masked sham-controlled study for the approval of pegcetacoplan, a complement C3 and C3b inhibitor, for the treatment of GA secondary to AMD.^8^ Patients over 60 years of age with BCVA >24 Early Treatment Diabetic Retinopathy Study (ETDRS) letters (20/320 Snellen equivalent) and a total GA area between ≥2.5 and ≤17.5 mm^2^ on FAF were included, as defined by the study protocol. MP was performed using the Macular Integrity Assessment (MAIA) MP (iCare, Centervue, SpA, Padova, Italy) in the mesopic standard setting (background luminance of 4 asb; 1.27 cd/m2; maximum intensity of 1000 asb, 36 decibel [dB] threshold dynamic range) by a fixed standardized 68 stimulus grid with a Goldmann III stimulus size corresponding to 0.43 degrees (=125 µm). According to the study protocol (clinicaltrials.gov, NCT03525613), the MP inclusion criteria were defined as follows: able to detect fixation target, total elapsed time to complete the 68 MP point exam takes below ≤30-minute duration, reliability test duration is ≤20%, as well as willingness to undertake the MP assessment.

This post hoc analysis was based on both study and fellow eyes from screening and baseline visits with matching MAIA examinations and Spectralis Spectral-domain (SD)-OCT (Spectralis HRA + OCT, Heidelberg Engineering, Heidelberg, Germany). SD-OCT volumes consisted of 49 B-scans of each 512 A-scans, covering the central 20 × 20 degrees of the macula with concurrent acquisition of a near-infrared reflectance (NIR) image covering a 30 × 30 degrees macular area. All screening failures were excluded from the study eye cohort, whereas the fellow eyes were manually graded for the presence of complete RPE and outer retinal atrophy (cRORA) based on the Classification of Atrophy Meetings (CAM) group criteria^5^ by a reader trained to reading center standards (author K.B.). All eyes included in this baseline analysis were still untreated eyes and did not receive any dose of pegcetacoplan at the time of this first MP examination.

Patients were included after informed written consent with review board approval by each participating center. Patient data were fully pseudonymized. The study adhered to the Declaration of Helsinki. This post hoc analysis was approved by the Ethics Committee at the Medical University of Vienna.

### OCT-Based Image Analysis and Point-Wise Co-Registration With Microperimetry

**Figure 1.**
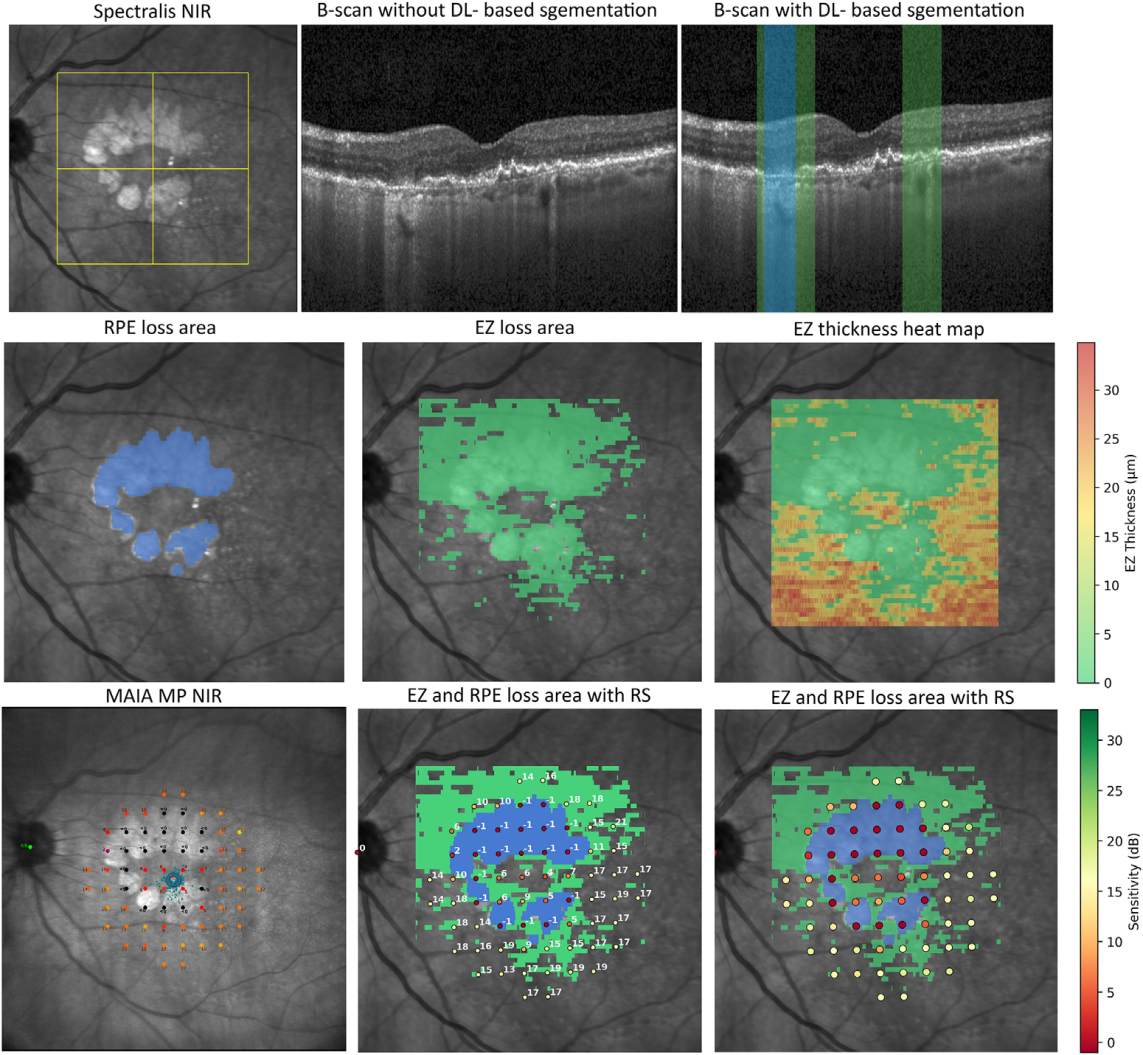
Correlation between deep learning–based OCT biomarkers and RS in dB*.* The OCT volume was acquired within a 20 × 20 degrees (6 × 6 mm) region (*yellow frame* on NIR in the *top row*) of the 30 × 30 degrees (9 × 9 mm) Spectralis OCT scan. RS values are spatially registered to the OCT volume and linked to the corresponding biomarker (EZ loss in *green* and RPE loss in *blue*). Reduced RS is observed within EZ and RPE loss areas. *Top row:* Spectralis NIR image and the corresponding central B-scan shown with and without automated segmentation of EZ (*green*) and RPE loss (*blue*). *Middle row:* En face maps of EZ and RPE loss area derived from volumetric segmentation in each respective B-scan, along with an EZ thickness heatmap (thickness scale ranging from 0–35 µm) derived from EZ thickness measurement in each B-scan. *Bottom row:* MAIA MP NIR image with the fixed 68-point test grid (*left*), the respective RS values in dB superimposed to the en face map of EZ and RPE loss area (*middle*), and a scale-based visualization of RS in dB (*right*). dB, decibel; DL, deep-learning; EZ, ellipsoid zone; MP, microperimetry; NIR, near infrared reflectance; OCT, optical coherence tomography; RPE, retinal pigment epithelium; RS, retinal sensitivity.

DL-based algorithms quantified EZ thickness, defined as the layer between the outer border of the interdigitation zone and the inner border of the EZ, and the EZ loss and RPE loss from OCT volumes (Fig. 1).^3^^,^^18^ En face EZ and RPE loss maps were derived for each volumetric OCT and defined as the respective areas of EZ and RPE loss.^19^ EZ thickness was automatically quantified outside of EZ and RPE loss and this region was defined as intact retina. Supplementary Figure S1 provides examples of automated biomarker quantification in OCT B-scans.

Registration between MP and OCT was performed within the 20 × 20 degrees area, where the OCT volume is acquired. In the MAIA MP, the maximum distance between 2 MP points was 9.06 degrees (approximately 2.7 mm), whereas the minimal distance between 2 neighboring MP points was 2 degrees (approximately 0.6 mm). Registration between the NIR of the MAIA MP and the Spectralis OCT was based on a previously published method.^12^^,^^20^ Registration algorithms superimposed the MAIA MP grid to the NIR of the Spectralis OCT based on the detection of corresponding landmarks of the retinal vasculature.^21^ The positions on the Spectralis NIR are already aligned to the respective B-scan, which enables a precise definition of the position for individual MP stimulus points at a B-scan level. Each MP stimulus point was allocated to the respective categories of EZ loss and RPE loss based on the center of each MP stimulus point, whereas EZ thickness was measured as the mean thickness in a 70-µm radius around each MP stimulus point. To avoid any discrepancies arising from the MP point allocation of the respective categories, MP points allocated to EZ and RPE loss with an EZ thickness measurement >0 µm within a 70-µm radius were excluded from the analysis. The percentage of RPE loss was calculated for each respective MP point as an additional parameter. Eccentricity was calculated for each MP point in millimeters (mm) and defined as the distance from the fovea based on manual fovea grading in all OCT volumes by two experienced readers (authors K.B. and M.L.E.). A subcohort was randomly selected to evaluate the accuracy of the semi-automated registration method. For this purpose, re-registration was performed by an experienced reader (author L.M.E.) based on manually defining identical anatomic landmarks visible in both imaging modalities (MAIA MP NIR and Spectralis OCT NIR). Using the least squares method, the affine transformation matrix was calculated and the stimulus positions were projected onto the NIR. Median registration deviation was calculated between MAIA MP NIR and the location on OCT B-scan, as well as between MAIA MP NIR and the location on the Spectralis OCT NIR. Fixation stability was provided for each MP examination and defined as stable, relatively stable, and unstable according to the commonly used classification.^22^

### Statistical Analysis

Descriptive statistics with mean and standard deviation were calculated for each variable. To investigate the association between RS and structural markers, a mixed effects model was used with the *lme* function from the *nlme* package in R software.^23^^,^^24^ This model included the following predictors: EZ thickness (as a continuous variable), EZ loss and RPE loss (categorical variable with intact retina as the reference level), and retinal eccentricity. Additionally, an interaction term between eccentricity and the loss group was incorporated. The EZ thickness variable was adjusted by mean-centering within the EZ and RPE loss variables. For the variable intact retina, the mean EZ thickness of 23.07 µm was subtracted from each measurement. In the EZ loss and RPE loss groups, where thickness was consistently recorded as zero, no subtraction was performed. We evaluated multicollinearity among the OCT predictors (EZ thickness and EZ and RPE loss) using an equivalent linear model for the fixed effects of RS for EZ thickness, EZ and RPE loss. Diagnostics showed variance inflation factor (VIF) = 1.00 for group-centered EZ thickness and adjusted generalized variance inflation factor (GVIF) = 1.00 for the EZ and RPE loss factor. The fixed-effects design condition number was κ = 19.58. These values indicate negligible collinearity (VIF ≈ 1–2 considered low; κ = <30 generally unproblematic). The use of group-centering for EZ thickness within EZ and RPE loss categories implies orthogonality to the factor main effect, consistent with VIF ≈ 1. No remedial action was required. The model included a nested random intercept structure to account for intra-patient variability, with measurements clustered within each patient and further nested by eye position (left and right). To allow for variation in the effect of loss group across different eye positions, random slopes were included. Maximum Likelihood (ML) estimation was utilized, facilitating model comparison and the use of likelihood ratio tests to evaluate the inclusion of fixed effects. The performance of the exploratory model was evaluated using both the marginal and conditional coefficients of determination (R^2^GLMM) for generalized mixed-effect models. The marginal *R^2^* quantifies the variance explained by the fixed effects alone, whereas the conditional *R^2^* accounts for both the fixed and random effects. Predictive accuracy was assessed through the correlation between predicted and observed RS. Additionally, a visualization plot displaying regression lines for EZ and RPE loss groups was created to visually assess model fit. The intra-class correlation coefficient (ICC) was also reported, quantifying the proportion of the total variance in the observed data that can be attributed to differences among patients and eye positions within them. An additional linear mixed-effect model was calculated for a subset analysis between MP points with EZ thickness >20 µm, EZ thickness ≤20 µm (partial EZ loss), and EZ thickness 0 µm (EZ loss). Pairwise group differences were assessed with Tukey's HSD post hoc test on the estimated marginal means. A *P* value of < 0.05 was considered nominally significant.

## Results

### Baseline Characteristics

MP examinations were successfully matched with Spectralis OCT imaging in 762 eyes of 406 patients. A total of 84 of 762 eyes were excluded from the analysis: 49 fellow eyes were excluded following manual grading for cRORA, and 35 study eyes were excluded due to screening failures, see detailed description in Supplementary Figure S2. The final analysis was performed on 41,925 MP stimuli from 678 eyes with GA secondary to AMD from 406 patients with 361 study eyes and 317 fellow eyes. From 46,104 available MP points (678 eyes × 68 MP stimulus point grid), 2.9% (1368/46,104) could not be matched to OCT biomarkers und were therefore excluded during the pointwise co-registration. Then, from 44,736 (46,104 − 1368 = 44,736) successfully matched MP points, 2811 MP points were excluded post hoc after pointwise co-registration to achieve the highest precision in point allocation (see Methods). Therefore, 41,925 MP points (46,104 − 1368 − 2811 = 41,925) were available for final analysis with 61% (25,713 / 41,925) located within intact retina, 10% (4271 / 41,925) located within EZ loss and 28% (11,941/ 41,925) within RPE loss. Registration accuracy was analyzed in a subset of 79 eyes (MP points = 5152) with a median registration deviation of 28.21 (interquartile range [IQR] = 18.66–46.81) µm between MAIA MP NIR and the location on OCT B-scan and 42.95 (IQR = 28.32–59.37) µm between MAIA MP NIR and Spectralis OCT NIR. Fixation stability showed that 58% (399/678) of MP points were stable (202/678, 29.79%) or relatively stable (197/678, 29.06%), whereas 41.15% (279/678) were unstable.

**Figure 2.**
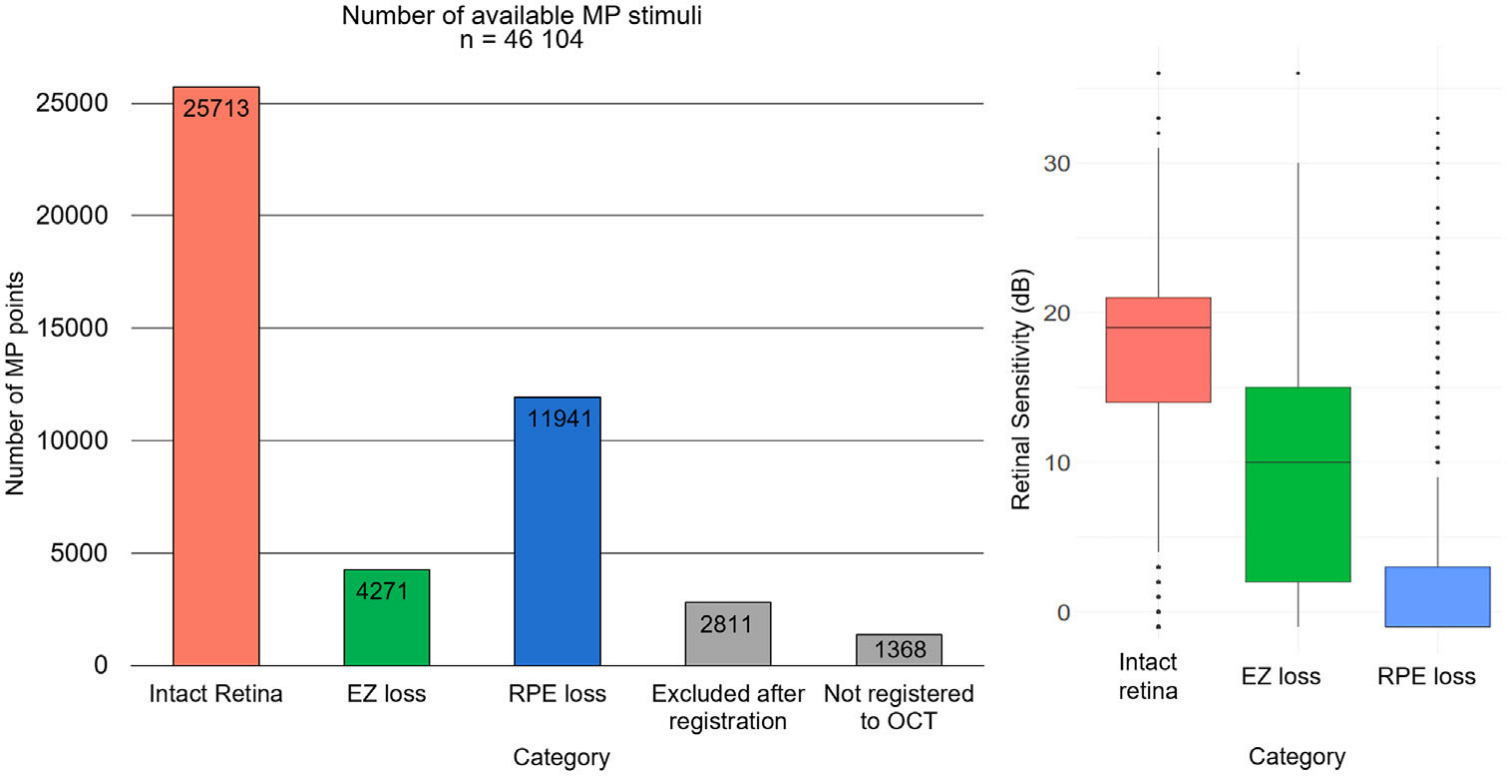
*Left:* Bar chart illustrating the total number of MP stimuli per category, including a breakdown of all exclusions, resulting in 41,925 MP stimuli available for final analysis: 25,713 within intact retina, 4271 within EZ loss, and 11,941 within RPE loss. *Right:* Boxplots showing retinal sensitivity values for each OCT category.

Overall mean RPE loss and EZ loss area were 6.88 ± 3.81 mm^2^ (minimum 0.32 mm^2^ and maximum 19.08 mm^2^) and 10.59 ± 5.34 mm^2^ (minimum 0.922 and maximum 26.92 mm^2^), respectively. Overall, mean RS was 11.87 ± 9.23 dB. Mean EZ thickness within intact retina was 23.07 ± 7.15 µm. Mean distance between fovea and MP points was 1.8 ± 0.7 mm (minimum 0 mm and maximum 4.07 mm). Figure 2 summarizes the descriptive statistics, indicating the number of points within the intact retina, EZ loss and RPE loss, and the respective RS. Sixty-one percent (25,713/41,925) of MP points were located within intact retina with a mean RS of 16.88 ± 6.7 dB, 10% (4271/41,925) within EZ loss with mean RS 9.14 ± 7.2 dB and 28% (11,941/41,925) within RPE loss with mean RS of 2 dB ± 5.6 dB.

### Correlation of Quantified Morphology and Sensitivity Values

The Table displays the results from the multivariable model. Increased EZ thickness improved RS by 0.2 dB/µm, whereas EZ loss and RPE loss presence reduced RS by −8 dB and −14 dB compared to intact retina, respectively (*P* < 0.001). The marginal *R^2^* was 0.44, whereas the conditional *R^2^* was 0.74 with correlations between predicted and observed RS of 0.88. A correlation plot for model accuracy is displayed in Supplementary Figure S3.

**Table.** Mixed Effect Model Identifying Correlations Among Retinal Sensitivity (dB) Changes and EZ Thickness, EZ Loss, and RPE Loss With Upper and Lower Limits Defined as 95% Confidence Intervals

Variable	Estimate	Upper Limit	Lower Limit	*P* Value
Intercept for intact retina	15.0 dB	15.6 dB	14.5 dB	<0.0001
EZ thickness	0.2 dB/µm	0.2 dB/µm	0.2 dB/µm	<0.0001
EZ loss presenceReference: no loss	−8.2 dB	−7.7 dB	−8.7 dB	<0.0001
RPE loss presenceReference: no loss	−14.4 dB	−14.9 dB	−14.9 dB	<0.0001
Eccentricity	0.3 dB/mm	0.2 dB/mm	0.4 dB/mm	<0.0001
EZ loss* eccentricity	1.7 dB	1.4 dB	1.9 dB	<0.0001
RPE loss* eccentricity	1.5 dB	1.3 dB	1.7 dB	<0.0001

dB, decibel; EZ, ellipsoid zone; RPE, retinal pigment epithelium.

The intercept is the predicted RS in areas with mean EZ thickness of 23.07 µm.

**Figure 3.**
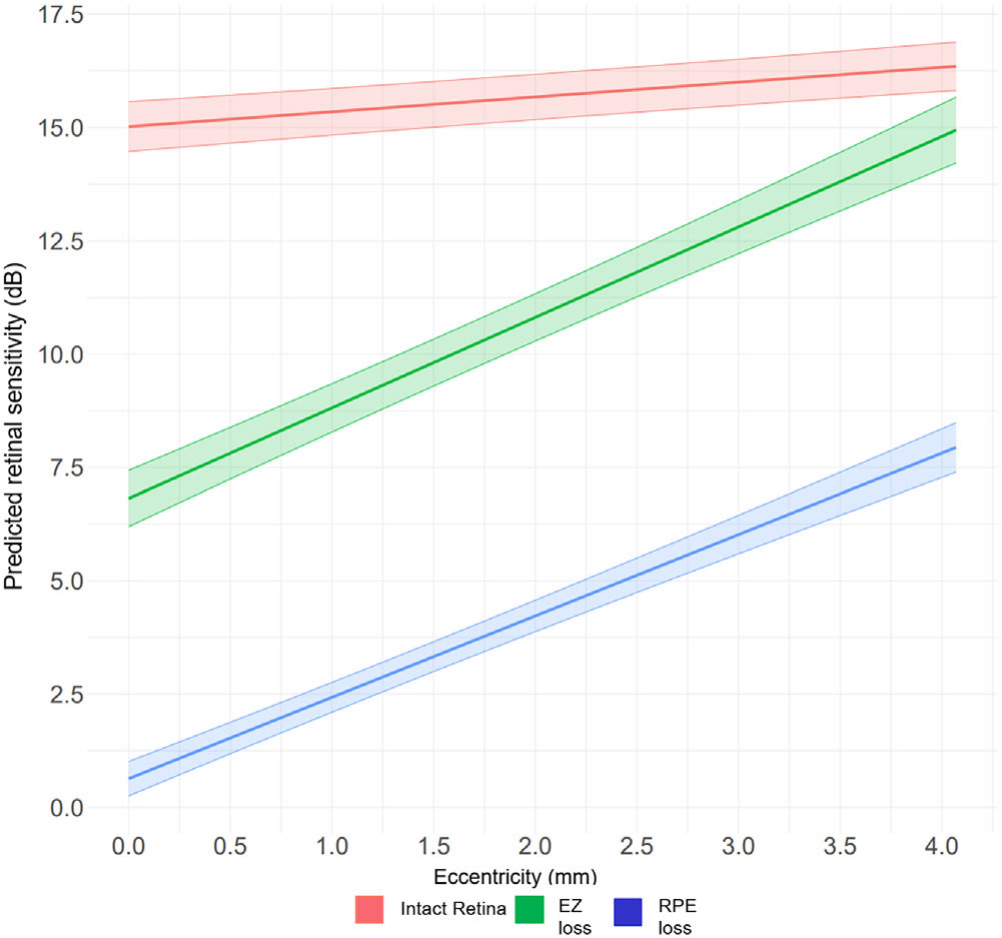
Interaction plot between the metric predictor retinal sensitivity within the factor OCT category (RPE loss, EZ loss, and intact retina) correlated to retinal eccentricity (mm).

**Figure 4.**
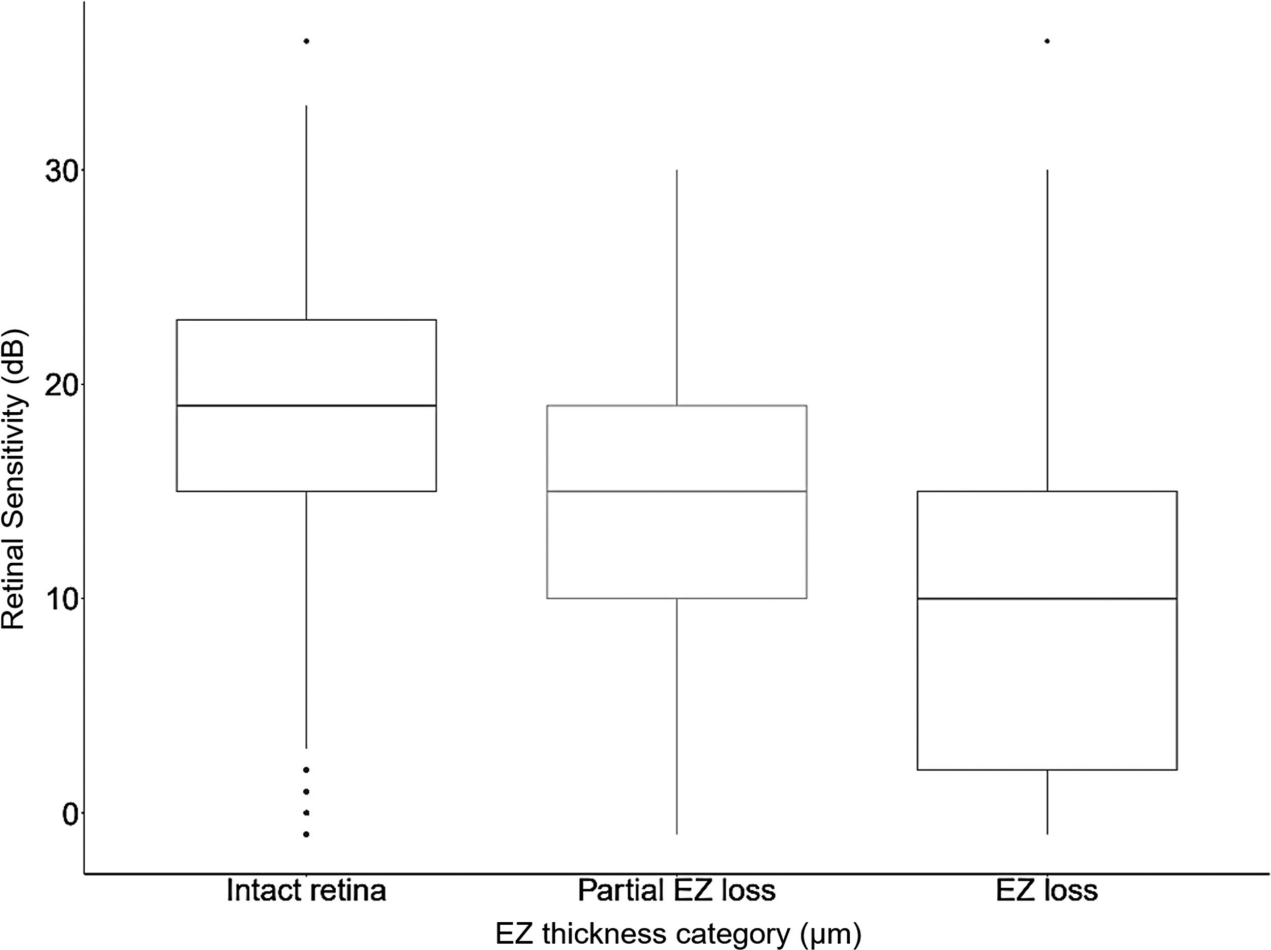
Box plots of retinal sensitivity in dB within intact retina (EZ thickness >20 µm), EZ thickness ≤20 µm (partial EZ loss) and EZ thickness 0 µm (EZ loss).

Figure 3 shows the interaction plot for intact retina, EZ loss and RPE loss, and retinal eccentricity. A positive interaction between RS and distance from fovea is attributed to 2/3 of subfoveal GA lesions in this cohort, as previously published.^8^ An exploratory subset analysis revealed a significant difference between MP points with EZ thickness >20 µm, EZ thickness ≤20 µm (partial EZ loss), and EZ thickness at 0 µm (EZ loss; *P* < 0.001), as presented in Figure 4 and Supplementary Table S1.

### Percentage of Scotomatous Points Within RPE Loss

A subset analysis of MP stimulus points located within the clinical GA lesion area, defined as RPE loss on OCT, was performed in 11,941/41,925 MP points. 69.98% (8356/11,941) presented a RS of 0 dB or −1 dB. There were 30.02% of MP points that exhibited an RS above 0 dB; 10.63% (1269/11,941) had an RS between 0 dB and 5 dB, 7.53% (899/11,941) between 5 dB and 10 dB, and 12% (1417/11,941) were above 10 dB. Additional analysis on the percentage of RPE loss area for each individual MP point showed that an increasing percentage of RPE loss area within each MP point was associated with lower RS measurements. Detailed results are summarized in Supplementary Table S2 and Supplementary Figure S4.

## Discussion

This study provides robust evidence on topographic RS decline in corresponding GA-related OCT biomarkers in a cohort consisting of 409 patients from a standardized clinical trial setting. Our approach fills the knowledge gap among morphological biomarkers, EZ and RPE loss on OCT, and their impact on functional decline in MP. This structure/function correlation offers pivotal insights into the monitoring of functional decline in GA in clinical practice and supports the definition of novel functional endpoints for clinical trials. Based on the large size of the population, the prospective OCT and MP performance and the standardized lesion characteristics, the results encompass a mere proof-of-principle, but establish for the first time a quantitative correlation of EZ thickness (µm) and RS (dB). Hence, enabling a reliable extrapolation from EZ alteration to the expected functional outcome.

Takahashi et al. assessed perilesional areas with photoreceptor damage defined as disruption of the EZ band based on manual SD-OCT grading in a small cohort and found that mean RS was 6.57 dB in areas with photoreceptor discontinuity, which is significantly lower compared with healthy areas.^25^ This finding is consistent with the estimate from our model, which predicts RS of 6.8 dB within areas of complete EZ loss. Our model indicates that once EZ thickness is 0 µm (corresponding to EZ loss), RS is reduced by −8 dB (95% confidence interval [CI] = 8–9 dB], which results in an estimated predicted RS within EZ loss of 6.57 dB. This is above the reported test-retest variability of ± 3 to 6 dB in MAIA in patients with GA.^14^^,^^26^ Concurrently, previously published work by our group on the methodology of pointwise registration between MP and DL-based OCT analysis also provided comparable estimates in these predefined areas.^12^ Importantly, model estimates are based on the dataset used for calculation. Still, it was reported previously that test-retest outcomes in MAIA increase in areas with EZ and RPE loss.^14^ Therefore, repeatability of RS measurements needs to be investigated further in larger cohorts to define appropriate RS thresholds for clinical trials. Nonetheless, the consistency of this finding across different MP grids and GA cohorts further validates our proof-of-concept results based on automatically quantified areas of EZ loss in a much larger cohort. It is obviously the quality of a fully automated approach applied to large data sets that lends reproducibility to the structure/function correlation, where function relies on a method, such as MP, with an intrinsically high variability. Providing robust evidence on the value of quantification of the EZ layer is the first step toward validating subclinical OCT biomarkers in GA. Based on this structure/function correlation, personalized quantification of EZ loss and EZ thickness is a promising OCT-based biomarker associated with retinal function in GA. Longitudinal analysis of RS and high-risk OCT biomarkers will allow us to determine the EZ thickness change over time and its impact on functional outcomes.

The area surrounding GA lesions, commonly defined as the junctional zone or perilesional area, remains the region of interest for definition of trial endpoints due to its representation of lesion growth, that is, disease activity.^13^^,^^15^ This area has previously been defined as a fixed distance of 250 µm surrounding the GA area. Pfau et al. showed reduced mean RS in this area,^27^ whereas a post hoc analysis of the Chroma and Spectri trial revealed a significant decrease in mean RS during follow-up in areas surrounding scotomas in MP.^15^ A recent post hoc analysis of the OAKS trial by Chakravarthy et al. revealed that patients treated with pegcetacoplan developed fewer areas of absolute scotomas within this junctional zone compared to sham.^13^ On OCT, GA lesions are surrounded by photoreceptor degeneration, which appears as loss of the EZ and can be quantified using DL algorithms.^28^ DL-based image analysis of the EZ loss areas, however, clearly indicates that advanced EZ thinning is focal and irregular as well as widely variable between patients.^29^ Vogl et al. showed that local progression was significantly higher for areas with a thinner EZ layer.^28^ Consequently, quantification and localization of the EZ loss area, as well as accurate EZ thickness, gained relevance in recent years for the prediction of GA growth rate and implications for therapeutic response.^29^ Importantly, the US Food and Drug Administration (FDA) approved EZ attenuation as a structural clinical trial endpoint and further improvements in understanding the EZ layer are called for in the current literature.^30^ We provide robust evidence on the functional relevance of EZ loss in general and thickness quantification, in particular, in OCT volumes, which is in line with current knowledge on the significant correlation between EZ status and visual function.^31^ Interestingly, the impact of EZ thickness on RS by 0.2 dB/µm is also a very consistent finding in previous work in patients with non-exudative AMD.^12^^,^^20^^,^^32^ The same estimates were found in patients with diabetic macular edema.^32^ Most importantly, according to previous literature, the effect of EZ thickness on RS remains robust in the presence of other morphological comorbidities, such as drusen,^12^^,^^20^ reticular pseudodrusen,^20^ hyper-reflective foci,^12^^,^^20^ and macular edema.^32^ Still, high-risk AMD biomarkers, including drusen, reticular pseudodrusen, and/or hyper-reflective foci, which might be located within intact retina, as defined by the present study, could impact localized RS.^12^^,^^20^^,^^33^ Interestingly, both EZ thickness and RS within intact retina are significantly lower compared with pointwise values in patients with intermediate AMD and healthy controls quantified with the same EZ thickness measurement algorithm.^20^^,^^34^ This highlights the global functional decline in patients with GA, even in areas with preserved EZ integrity and the importance of EZ thickness quantification as a potential outcome measure. The linear correlation between EZ thickness and function has great implications for the development of functional trial endpoints, as the quantification of EZ thickness alone may allow for an individualized prediction of localized sensitivity changes by each µm of EZ thickness change. Concurrent approaches to dichotomize the variable into partial and complete EZ loss and/or EZ attenuation further highlights this statistical relationship (see Fig. 4). Still, the potential statistical error from dichotomization of a metric variable and concurrent information loss needs to be considered.^35^^–^^37^

Analysis of the evolution of scotomatous points is another increasingly recognized direction in current research.^11^^,^^13^^,^^15^ Chakravarthy et al. found that the progression of the four central macular MP points to absolute scotomas occurred with lower probability in patients undergoing treatment with pegcetacoplan.^13^ Interestingly, a direct correlation between BCVA and scotomas within the central 4 and 16 MP points was reported.^13^ We provide evidence of remaining RS in 30% of MP points located within areas of RPE loss on OCT, which correlate to FAF measurements, as proven previously.^6^ This result is in line with previous work by Pfau et al., who found evidence for remaining light sensitivity within areas of complete RPE loss.^27^ This presumed discrepancy between structure and function within areas of RPE loss on OCT could be linked to the spot size of the MP stimulus point on the retina with light scattering to surrounding intact tissue or to preserved secondary neurosensory activity. We further calculated the percentage of the RPE loss area for each individual MP point, which directly influences RS of the respective stimulus. This reveals that the condition of the retina can differ within each MP point and directly influences RS.

Increasing retinal eccentricity is physiologically associated with a decrease in RS in healthy aging eyes.^34^^,^^38^ The inversion of this effect in our cohort (see Fig. 3^4^) is attributed to a higher number of eyes presenting with foveal GA with the lowest RS in the central macula. Higher RS with increasing eccentricity within intact retina could be influenced by the mesopic setting with lower stimulation of the foveal cones compared with the parafoveal rods and cones. Previously published structure/function correlation in a smaller GA cohort revealed reduced RS with greater distance from the foveal center in a different MP grid, which included a different distribution of MP points compared to the grid used in the OAKS trials for which we corrected by manual annotation of the fovea.^12^^,^^34^

The strength of our analysis is the implementation of an established method of pointwise co-registration between MP and OCT in a large study population for robust proof-of-concept. Our work has limitations that should be considered for the interpretation of the results. First, the retrospective nature of this analysis poses a limitation for determination of sample size. Second, despite a thorough process and manual quality control by certified experts, minimal deviations in the location of the MP point on OCT after the registration process are inevitable, which were calculated by providing the registration error. This likely contributes to outliers with higher RS values within the RPE loss groups. Third, the use of a standard grid limits the number of MP points within EZ and RPE loss areas. Furthermore, standard MP grids might under-represent key high-risk structural features, affecting the calculated RS estimates within the areas of EZ and RPE loss.^13^ The use of a lesion-targeted, individualized method (Tratnig-Frankl, IOVS, 2025, Volume 66, ARVO E-abstract), as well as focal grids focusing on personalized lesion components could be a future MP testing direction for clinical trials.^16^ Still, the large amount of MP points within intact retina allows for calculation of robust estimates of EZ thickness. Whether partial EZ loss represents a robust parameter remains to be evaluated regarding various other morphological contributors such as drusen and pseudodrusen, as well as image quality. Complete EZ loss might offer superior reliability and reproducibility and can be introduced into clinical practice with the availability of artificial intelligence (AI)-based tools with regulatory approval in Europe.

In conclusion, MP is a well-suited visual function test to objectively assess manifest functional loss in the macular region, which is correlated to defined areas and grades of morphological photoreceptor degeneration on OCT. Quantification of EZ thickness allows for personalized prediction of RS, both for individual GA lesions as well as for individual patients. The direct association between RS by dB in MP and photoreceptor thinning in µm on OCT, as automatically quantified in clinical GA, together with visualization of high-risk areas of EZ thinning and loss, provides essential tools for establishing functional and morphological trial endpoints and may guide the clinical management of GA in the near future.

## Supplementary Material

Supplement 1
