# Supplementary material for: Exploring Trial Endpoints in Geographic Atrophy Based on Localized Functional Changes in Microperimetry and AI-Quantified OCT Biomarkers
Source: Invest Ophthalmol Vis Sci. 2026 Jan 9;67(1):22. doi: 10.1167/iovs.67.1.22 (PMC12805968; doi:10.1167/iovs.67.1.22)
Supplement: Supplement 1 [file iovs-67-1-22_s001.docx]

**Supplementary material**

**
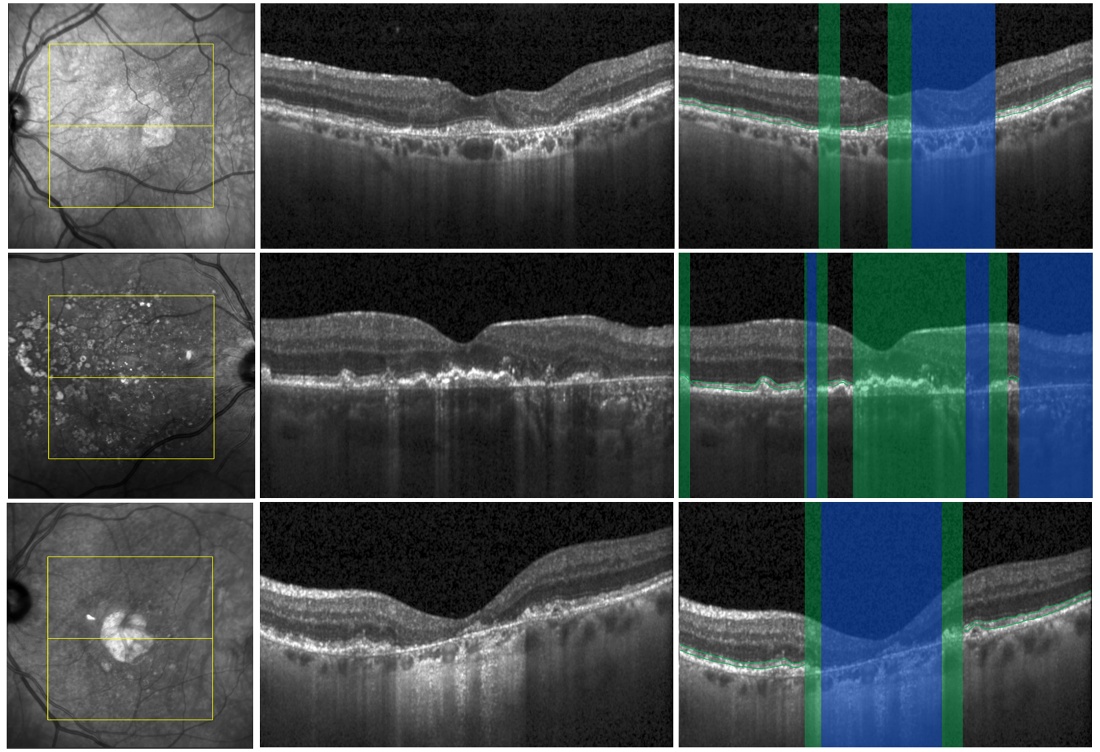
**

**Supplementary Figure 1:** Examples of automated DL-based quantification of EZ thickness between the outer border of the interdigitation zone and the inner border of the ellipsoid zone (green lines), EZ loss defined as loss of the EZ layer (EZ thickness 0 µm) marked in green and RPE loss defined as loss of the RPE layer marked in blue in OCT B-scans. All three examples show different morphological GA characteristics. The upper row shows parafoveal RPE loss and a smaller area of EZ loss, the example in the middle row shows calcified drusen with a larger area of EZ loss and the example in the lower row shows a large area of central RPE loss. EZ = ellipsoid zone, RPE = retinal pigment epithelium, GA = geographic atrophy, OCT = optical coherence tomography, DL = deep-learning

Assessed for eligibility for study inclusion***** (patients = 1325)

1145 MP examinations (eyes = 364, patients = 185) excluded

- MP examinations excluded due to imaging by another vendor (Cirrus or Topcon) = 832
- MP examinations excluded due to withdrawn consent = 120
- MP examinations excluded due to missing OCT scan = 94
- MP examinations excluded due to no matching between OCT and MP = 87
- MP examinations excluded due to low quality = 12

4013 MP examinations (eyes = 1249, patients = 647) were available at any timepoint (baseline or follow-up)

2868 MP examinations (eyes = 885, patients = 462) successfully matched between Spectralis OCT and MAIA MP

762 MP examinations (eyes = 762, study eyes = 396, fellow eyes = 366; patients = 406) successfully matched at baseline visit

2106 MP examinations (eyes = 123, patients = 56) performed during follow-up visits excluded

84 MP examinations, eyes = 84

excluded during data-cleaning

- Excluded in the study eye cohort due to screening failure eyes = 35
- Excluded in fellow eyes cohort after manual grading of OCT volumes for presence of cRORA with exclusion of eyes with intermediate or neovascular AMD eyes = 49

678 MP examinations (eyes = 678, study eyes = 361, fellow eyes = 317; patients = 406)

**Supplementary Figure 2:** Consort flow chart on selection of retrospective patient cohort based on available MP MAIA exams and Spectralis OCT imaging. *The initial number of patients is derived from Figure 1 trial profile to display all eyes screened for study inclusion from publication by Heier et al., Lancet 2023 (Heier et al., 2023; Heier, J. S., Lad, E. M., Holz, F. G., Rosenfeld, P. J., Guymer, R. H., Boyer, D., Grossi, F., Baumal, C. R., Metlapally, R., Deschatelets, P., Francois, C., Bliss, C., Ribeiro, R., Korobelnik, J. F., Korobelnik, J. F., Slakter, J. S., Waheed, N. K., Pearce, I., Steinle, N., … Dubska, Z. (2023). Pegcetacoplan for the treatment of geographic atrophy secondary to age-related macular degeneration (OAKS and DERBY): two multicentre, randomised, double-masked, sham-controlled, phase 3 trials. The Lancet, 402(10411), 1434–1448. https://doi.org/10.1016/S0140-6736(23)01520-9). MP = Microperimetry, OCT = optical coherence tomography

**
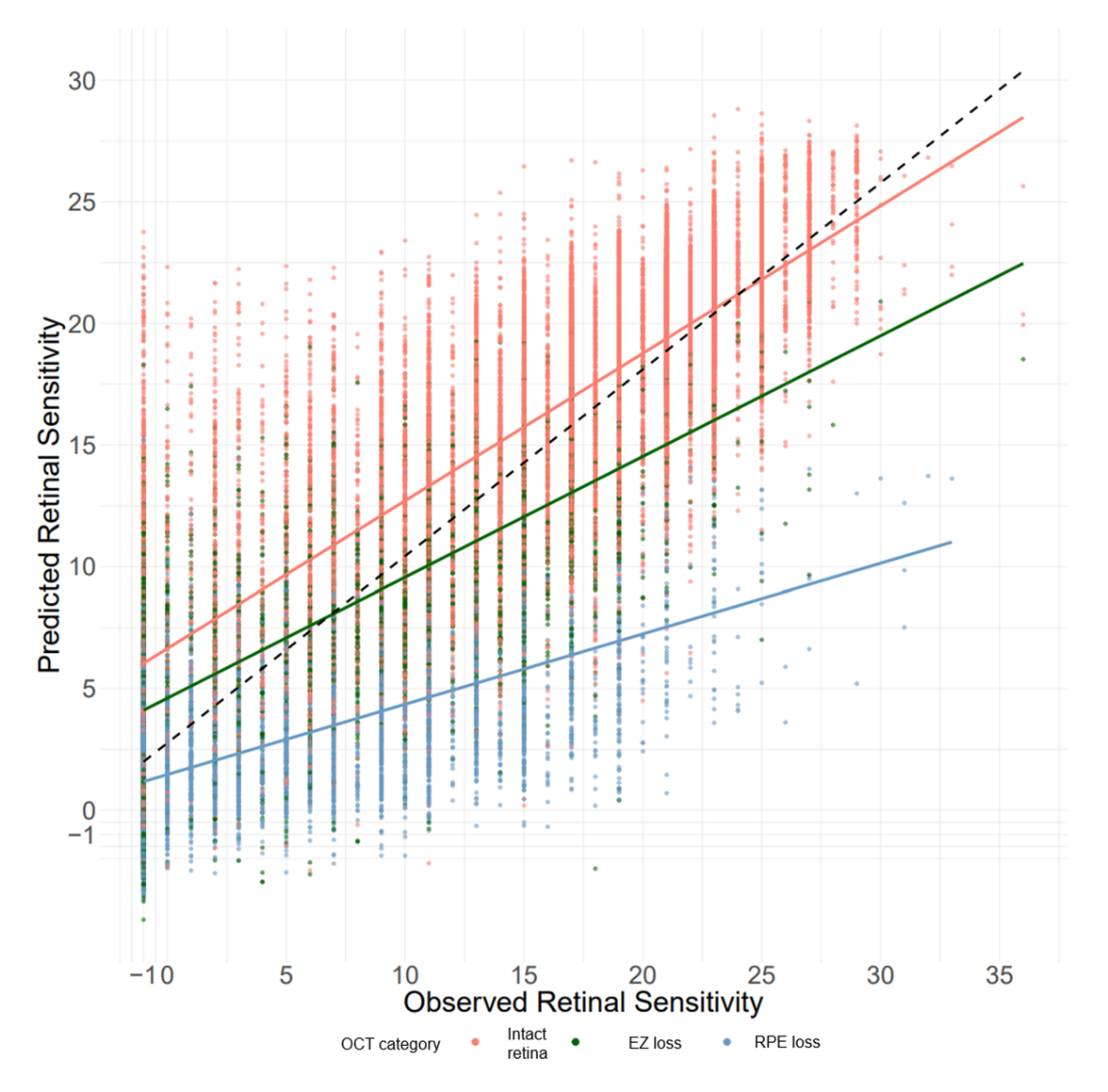
**

**Supplementary Figure 3:** Observed and predicted retinal sensitivity (dB) by each OCT category. EZ = ellipsoid zone, RPE = retinal pigment epithelium, dB = decibel

| **EZ category** | **Estimated mean RS** | **Upper limit** | **Lower limit** |  |
| --- | --- | --- | --- | --- |
| Intact retina  EZ thickness > 20 µm | 16.43 dB | 16.95 dB | 15.91 dB |  |
| EZ thickness ≤ 20 µm | 13.80 dB | 14.30 dB | 13.29 dB |  |
| EZ loss  EZ thickness = 0 µm | 9.93 dB | 10.42 dB | 9.44 dB |  |
| **Pairwise comparison** | **Estimated mean RS difference** | **Upper limit** | **Lower limit** | **p value** |
| Intact retina –  EZ thickness ≤ 20 µm | 2.63 dB | 2.90 dB | 2.37 dB | *<0.0001* |
| Intact retina –  EZ loss | 6.50 dB | 6.95 dB | 6.05 dB | *<0.0001* |
| EZ thickness ≤ 20 µm –  EZ loss | 3.86 dB | 4.22 dB | 3.50 dB | *<0.0001* |

**Supplementary Table 1:** Linear mixed effect model with pairwise comparison between groups of EZ thickness. Upper and lower limit defined as 95% confidence interval. EZ = ellipsoid zone, dB = decibel, RS = retinal sensitivity


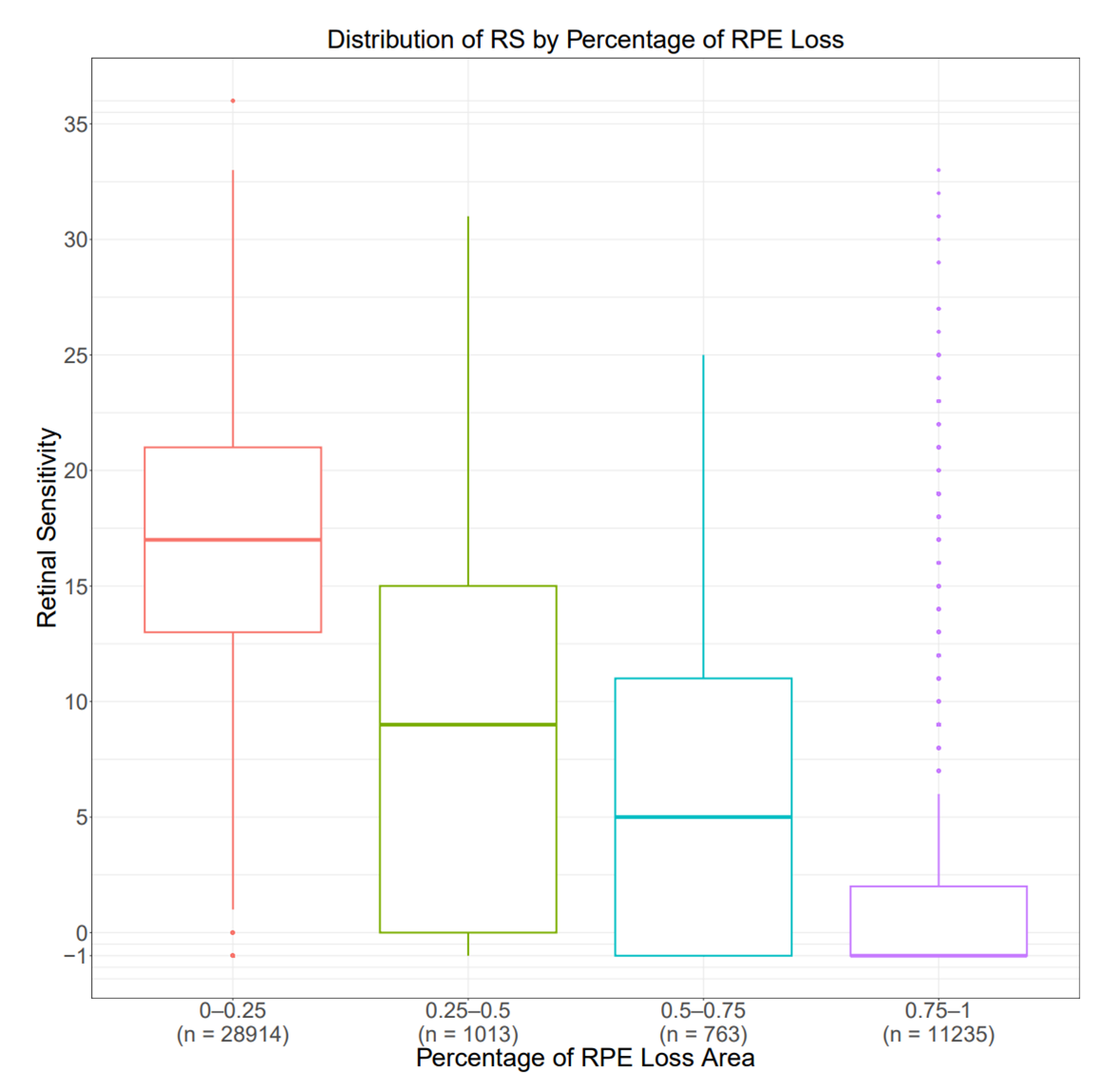


**Supplementary Figure 4:** Boxplots of retinal sensitivity (dB) for percentage of RPE loss area within each respective MP stimulus point. RPE = Retinal pigment epithelium, RS = retinal sensitivity, dB = decibel

| Percentage of  RPE loss | 0–25%  (n = 28914/ 41925) | 25–50%  (n = 1013/ 41925) | 50–75%  (n = 763/ 41925) | 75–100%  (n = 11235/ 41925) |
| --- | --- | --- | --- | --- |
| Number of MP points categorized as RPE loss based on centerpoint | 0 (0 %) | 89 (8.79 %) | 620 (81.26 %) | 11232 (99.97 %) |
| Mean RS | 16.06 ± 7.12 dB | 8.43 ± 7.73 dB | 5.93 ± 6.92 dB | 1.81 ± 5.4 dB |

**Supplementary Table 2:** Percentage of RPE loss and the respective retinal sensitivity (dB) for each individual MP point. RPE = retinal pigment epithelium, dB = decibel
